# Prolonged androgen deprivation leads to downregulation of androgen receptor and prostate-specific membrane antigen in prostate cancer cells

**DOI:** 10.3892/ijo.2012.1649

**Published:** 2012-10-04

**Authors:** TIANCHENG LIU, LISA Y. WU, MELODY D. FULTON, JACQUELINE M. JOHNSON, CLIFFORD E. BERKMAN

**Affiliations:** Department of Chemistry, Washington State University, Pullman, WA 99164, USA

**Keywords:** androgen receptor, prostate-specific membrane antigen, androgen deprivation, prostate cancer

## Abstract

Emergence of androgen-independent cancer cells during androgen deprivation therapy presents a significant challenge to successful treatment outcomes in prostate cancer. Elucidating the role of androgen deprivation in the transition from an androgen-dependent to an androgen-independent state may enable the development of more effective therapeutic strategies against prostate cancer. Herein, we describe an *in vitro* model for assessing the effects of continuous androgen-deprivation on prostate cancer cells (LNCaP) with respect to the expression of two prostate-specific markers: the androgen receptor (AR) and prostate-specific membrane antigen (PSMA). Compared with androgen-containing normal growth medium, androgen-deprived medium apparently induced the concomitant downregulation of AR and PSMA over time. Decreased protein levels were confirmed by fluorescence imaging, western blotting and enzymatic activity studies. In contrast to the current understanding of AR and PSMA in prostate cancer progression, our data demonstrated that androgen-deprivation induced a decrease in AR and PSMA levels in androgen-sensitive LNCaP cells, which may be associated with the development of more aggressive disease-state following androgen deprivation therapy.

## Introduction

Prostate cancer remains the second leading cause of cancer death for Northern American men. According to the National Cancer Institute, it is estimated that there will be 241,740 new cases and 28,170 deaths from prostate cancer in the United States in 2012 (http://www.cancer.gov/cancertopics/types/prostate). Initially, prostate cancer cells depend on androgen stimulation for growth and proliferation, and sensitive to androgen deprivation therapy. Unfortunately most recurrent tumors return within two years with an androgen-independent state and an aggressive, metastatic phenotype. As of yet there is no effective treatment for hormone-refractory prostate cancer (1,2).

Androgens and the androgen receptor (AR) are critical components that govern the development, differentiation, and functionality of the prostate gland and accessory reproductive tissues, and are also involved in prostate tumorigenesis (3). These pathological functions are dependent on the androgen-activated androgen receptor, which can act as a transcription factor to regulate the expression of multiple genes. Recently, several possible mechanisms (4,5) have been proposed for the progression of androgen-independent prostate cancer during androgen-deprivation therapy. Of them, mutation, amplification (5,6), and expression alternative-splice variants (7) of the androgen receptor are related to the adaptation of recurrent prostate cancer to low levels of androgen during androgen-deprivation therapy. Androgen deprivation also induces neuroendocrine differentiation (8) of prostate cancer cells leading to autocrine/paracrine signaling pathways for survival. In addition, androgen-independent prostate cancer cells have been characterized to exhibit stem cell-like properties (9,10).

Prostate-specific membrane antigen (PSMA), a type II membrane protein, has been found to be upregulated and strongly expressed on prostate tumor cells and as a consequence, it has attracted significant attention not only as a tumor marker for disease progression but also as both an imaging and therapeutic target for prostate cancer (11). Although previous studies revealed that PSMA expression was negatively regulated by androgen stimulation in AR-positive cells (12–14) and downregulation of AR expression was mediated by androgen-deprivation in an established androgen-independent subline derived from the androgen-sensitive LNCaP cell line (4), there are no reports which investigate the expression of both AR and PSMA at the protein level after long-term androgen deprivation. Therefore, this study was designed to identify the effect of long-term androgen-deprived growth on these two prostate cancer markers with respect to possible implications on diagnostic and therapeutic strategies for the androgen-independent disease-state. First, androgen-sensitive LNCaP cells were cultured in androgen depleted media up to 20 passages. Second, the altered expression of PSMA and AR at the protein levels induced by androgen deprivation was determined by western blotting, fluorescence imaging analysis, and enzymatic activity. Our data suggest that long-term androgen deprivation may induce the concomitant time-dependent downregulation of both AR and PSMA to progress toward a more aggressive, androgen-independent prostate cancer disease state.

## Materials and methods

### Cell lines and reagents

The human prostate cancer cell lines LNCaP and PC-3 were obtained from the American Type Culture Collection (Manassas, VA, USA). The rabbit polyclonal anti-AR antibody (N-20) was obtained from Santa Cruz Biotechnology, Inc. (Santa Cruz, CA, USA). The goat anti-rabbit secondary antibody-FITC and the mouse monoclonal anti-GAPDH antibody (clone GAPDH-71.1) were obtained from Sigma-Aldrich (St. Louis, MO, USA). The mouse monoclonal anti-PSMA antibody 7E11 was graciously provided by Cytogen Corp. (Princeton, NJ, USA). Protein blocking solution was obtained from BioGenex (San Ramon, CA, USA). Hoechst 33342 was obtained from Invitrogen-Molecular Probes (Carlsbad, CA, USA). Cy5.5-CTT-54.2 was prepared by our lab as described previously (15). Halt Protease Inhibitor Cocktail (100X) was purchased from Thermo Fisher Scientific (Rockford, IL, USA). All other chemicals and cell-culture reagents were purchased from Fisher Scientific (Sommerville, NJ, USA) or Sigma-Aldrich.

### Cell culture

LNCaP and PC-3 cells were grown in T-75 flasks with normal growth media [RPMI-1640 containing 10% heat-inactivated fetal calf serum (FBS), 100 U of penicillin and 100 *μ*g/ml streptomycin] in a humidified incubator at 37°C with 5% CO_2_. Otherwise, for androgen-deprivation growth, cells were cultured with conditioned media [RPMI-1640 containing 10% charcoal-stripped fetal bovine serum, 100 U of penicillin and 100 *μ*g/ml streptomycin]. Confluent cells were detached with a 0.25% trypsin 0.53 mM EDTA solution, harvested, and plated in two-well slide chambers at a density of 4×10^4^ cells/well. Cells were grown for three days before conducting the following experiments.

### Immunofluorescence detection of AR

The LNCaP cells, grown under androgen deprivation condition for over time (5, 10 and 20 passages), were cultured for 3 days on the slides in the conditioned media. For 2-day androgen-deprivation treatment, LNCaP cells were seeded on slides with normal growth media for 1-day growth, and replaced with conditioned media for another 2-day growth. Normal LNCaP cells and PC-3 cells were used for the AR-positive and AR-negative control respectively. These cells were seeded on slides with normal growth media for 3 days. Slides with 3-day growth cells in normal growth media or conditioned media were washed twice in PBS buffer (phosphate buffered saline), fixed in 4% paraformaldehyde in PBS buffer for 15 min at room temperature, and permeabilized with 0.2% Triton X-100 in PBS buffer for 5 min at room temperature. The permeabilized cells were blocked in block buffer (0.1% Tween-20, 5% goat normal serum in PBS buffer) for 2 h at room temperature and incubated with primary anti-AR antibody (100X diluted in block buffer) overnight at 4°C. After washing, the cells were incubated with a secondary antibody (goat anti-rabbit IgG-FITC, 40X diluted in 1% BSA, PBS buffer) for 2 h at room temperature, counterstained with Hoechst 33342, and mounted in Vectashield^®^ Mounting Medium (Vector Laboratories, Inc., Burlingame, CA, USA) for confocal microscopy.

### Affinity imaging of PSMAs with Cy5.5-CTT-54.2

The cells cultured on the 2-well slides were washed twice with warm medium (37°C) A (phosphate-free RPMI-1640 containing 1% FBS), then incubated with 1 ml of Cy5.5-CTT-54.2 (10 *μ*M) in warm medium A for 1 h in a humidified incubator at 37°C and 5% CO_2_. The above treated cells were washed three times with cold-KRB buffer pH 7.4 (mmol/l: NaCl 154.0, KCl 5.0, CaCl_2_ 2.0, MgCl_2_ 1.0, HEPES 5.0, D-glucose 5.0) and fixed with 4% paraformaldehyde in KRB for 15 min at room temperature. The cellular nuclei were counterstained with Hoechst 33342, and then mounted in Vectashield Mounting Medium for microscopy.

### Confocal laser scanning microscopy

Cells were visualized under 40× oil immersion objective using an LSM 510 META laser scanning microscope. Hoechst 33342 was excited with a Diode laser (405 nm), and the emission was collected with a BP420-480-nm filter. AR immunofluorescence (with goat anti-rabbit IgG-FITC) was excited at 488 nm using an Argon laser, and the emission was collected with a LP505-nm filter. PSMA-targeted imaging with Cy5.5-CTT-54.2 was excited using 633 nm from a HeNe laser, and the emission collected with an LP 650-nm filter. To reduce interchannel crosstalk, a multi-tracking technique was used, and images were taken at a resolution of 1,024×1,024 pixels. Confocal scanning parameters were set up so that the control cells without treatment did not have background fluorescence. The imaging colors of the fluorescent dyes, Hoechst 33342 and FITC, were defined as blue and green, respectively. As the emission wavelength of the near-infrared fluorescent dye Cy5.5 was beyond visible ranges, fluorescence pseudocolor of Cy5.5 was assigned as red. The images were edited by National Institutes of Health (NIH) Image J software (http://rsb.info.nih.gov/ij) and Adobe Photoshop CS2.

### Whole cell lysate extraction and western blot analysis

The controls: PC-3 and LNCaP cells (cultured in normal growth media) and LNCaP cells under androgen deprivation over time (2 days, 5, 10 or 20 passages) were collected by scraping, washed once in ice-cold PBS, re-suspended in 3-fold cell pellet volumes of lysis buffer (1% NP-40, 20 mM Tris pH 8.0, 137 mM NaCl, 10% glycerol) (16,17) supplemented with 1X Halt Protease Inhibitor Cocktail for 15 min on ice, then transferred to Eppendorf tubes for centrifugation at 10,000 g for 15 min at 4°C, the supernatant was saved as whole-cell protein extracts. Protein concentrations were determined using Non-Interfering Protein Assay (G-Biosciences, St. Louis, MO, USA). Western blotting was performed as described previously with only minor modifications (17,18). In brief, detergent soluble proteins (30 *μ*g) were loaded and separated on a NuPAGE™ 4–12% Bis-Tris Gel (Invitrogen, Carlsbad, CA, USA) by electrophoresis for 40 min at a constant 200 V under reducing conditions, and then transferred to a 0.45 *μ*m PVDF Immobilon-P Transfer Membrane (Millipore Corp., Bedford, MA, USA) at 400 mA for 100 min in a transfer apparatus-Owl Bandit VEP-2 (Owl, Portsmouth, NH, USA) according to the manufacturer’s instructions. Membranes were incubated with primary antibody at corresponding dilution overnight at 4°C and then with horseradish peroxidase conjugated-second antibody for 1 h at room temperature. The immunoreactive bands were visualized using Protein Detector TMB Western Blot kit (KPL, Gaithersburg, MD, USA) following the manufacturer’s instructions.

### Evaluation of PSMA relative enzymatic activity

HPLC-based PSMA enzymatic activity analysis was performed in triplicate as described previously with only minor modifications (19,20). Working solutions of the substrate (N-[4-(phenylazo)-benzoyl]-glutamyl-γ-glutamic acid, PABGgG) were made at 10 *μ*M in Tris-buffer (50 mM, pH 7.4). Working solutions of each protein sample were diluted at 30 *μ*g/ml in Tris-buffer (50 mM, pH 7.4 containing 1% Triton X-100). A typical incubation mixture (final volume 250 *μ*l) was prepared by the addition of 200 *μ*l Tris buffer (50 mM, pH 7.4) and PAB-Glu-γ-Glu (25 *μ*l, 10 *μ*M) in a test tube. The enzymatic reaction was initiated by the addition of 25 *μ*l of the PSMA working solution. The reaction was allowed to proceed for 15 min with constant shaking at 37°C and terminated by the addition of 25 *μ*l methanolic TFA (2.5% trifluoroacetic acid by volume in methanol) followed by vortexing. The quenched incubation mixture was quickly buffered by the addition of 25 *μ*l K_2_HPO_4_ (0.1 M), vortexed, iced for 15 min, followed by centrifugation (10 min at 7,000 g). An 85-*μ*l aliquot of the resulting supernatant was subsequently quantified for the contents of substrate and product by HPLC as previously described (21,22). Enzymatic activity for each protein sample was calculated from HPLC data. Activities were compared with normally-cultured LNCaP cells to evaluate relative enzymatic activity (REA).

## Results

### Downregulation of AR protein expression induced by androgen deprivation

**Figure 1:**
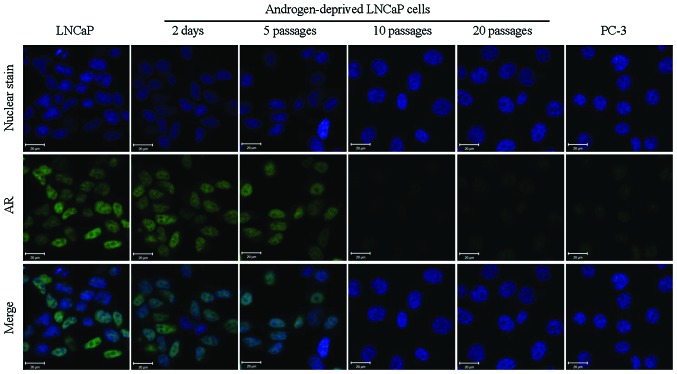
Downregulation of AR expression in LNCaP cells following androgen deprivation treatment. LNCaP cells, cultured in normal growth media, serve as positive controls. Strong fluorescence signals of AR were detected through the nuclei. The decreased signals were found in LNCaP cells treated with androgen deprivation for 2 days and 5 passages. There were no signals for 10 passages, 20 passages treated LNCaP cells, and AR-negative PC-3 cells. The nuclei were counterstained with Hoechst 33342 (blue). The cellular imaging was visualized by confocal microscopy; distance scale is 20 *μ*m.

In a positive control, a strong immunofluorescence signal for AR was observed throughout the nuclei of normal growth medium-cultured LNCaP cells (Fig. 1). In contrast, there is no signal for AR in AR-negative PC-3 cells (Fig. 1). Compared to the positive control, androgen deprivation induced downregulation of AR protein expression in LNCaP cells over time; AR was not detectable after 10 passages (Fig. 1). Based on these results, it is clear that downregulation of AR protein expression is a cell-adaptable response to prolonged androgen deprivation. Although the presented results were not consistent with the previous report which demonstrated that the administration of androgens resulted in downregulated AR mRNA levels in LNCaP cells (23), our data matched with androgen-independent LNCaP subline (4).

### Affinity labeling of PSMA

**Figure 2:**
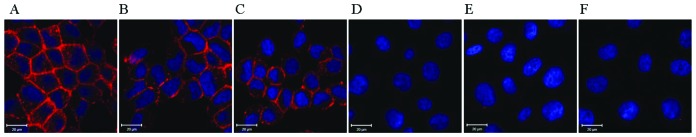
Downregulation of PSMA expression in androgen-deprived LNCaP cells by PSMA’s affinity labeling agent Cy5.5-CTT-54.2. (A) LNCaP cells cultured in normal growth media, serve as positive controls. The following LNCaP cells were treated with androgen deprivation for 2 days (B). (C) Five passages. (D) Ten passages. (E) Twenty passages. (F) PSMA-negative PC-3 cells. The nuclei were counterstained with Hoechst 33342. The cellular imaging was visualized by confocal microscopy; distance scale is 20 *μ*m.

Cy5.5-CTT-54.2, a specific PSMA fluorescent inhibitor (IC_50_ = 0.55 nM) was designed and evaluated for PSMA-targeted fluorescence imaging of LNCaP cells in our previous published study (15). In this study, Cy5.5-CTT-54.2 was employed to detect the change of active PSMAs on the cellular surface by fluorescence imaging of androgen-deprived LNCaP cells. Consistent with the results for AR immunofluorescence study above, the greatest cell labeling by Cy5.5-CTT-54.2 was observed for LNCaP cells cultured in normal growth media (Fig. 2A), with decreased signals through 5 passages under androgen-deprived conditions (Fig. 2B and C), and no detectable signals by 10 passages under the same conditions (Fig. 2D and E), similar to that of PSMA-negative PC-3 cells (Fig. 2F).

### Western blot analysis

**Figure 3:**
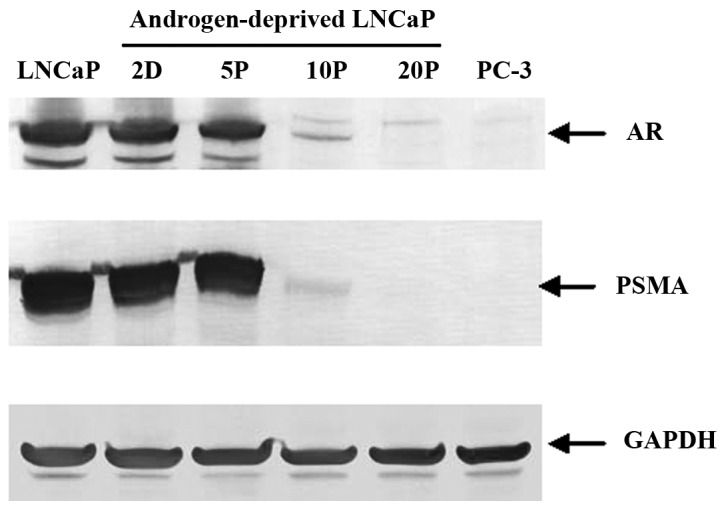
AR and PSMA expression were detected by western blotting. PSMA expression was downregulated in LNCaP cells treated with androgen deprivation for 2 days (2D) and 5 passages (5P), and very weak expression in cells treated for 10 passages (10P), completely undetectable in cells treated for 20 passages (20P). LNCaP cells cultured in normal growth media, serve as PSMA-positive controls, and PC-3 cells are designed as negative controls. GAPDH expression was detected to serve as a protein loading controls.

Western blot analysis further confirmed that the total amount of AR and PSMA decreased over time with a dramatic loss by 10 passages and absent after 20 passages in androgen-deprived conditions; GAPDH served as a protein loading controls (Fig. 3). These data suggest that downregulation of both AR and PSMA expression is dependent on the androgen levels and the length of time of androgen deprivation during cell growth.

### PSMA relative enzymatic activity (REA)

**Figure 4:**
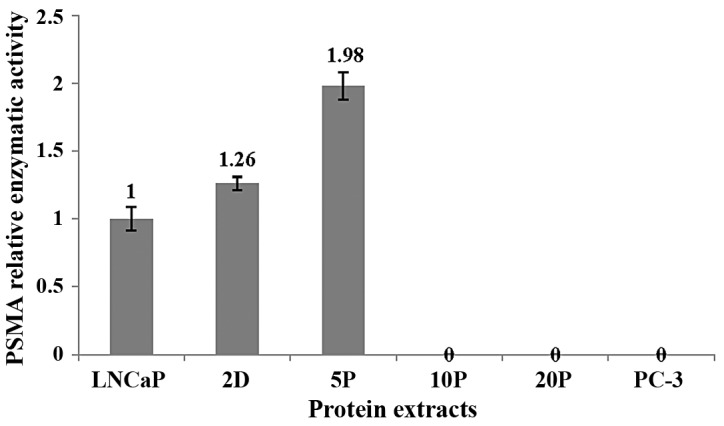
PSMA relative enzymatic activity (REA) increased for the protein extracts from short-term androgen deprived LNCaP cells: 2 days (REA=1.26) and 5 passages (REA=1.98). No detectable PSMA enzymatic activity observed for the protein extracts from long-term androgen deprived LNCaP cells (10 and 20 passages), similar to that of PSMA-negative PC-3 cells. PSMA enzymatic activity of the protein extract, from LNCaP cells cultured in normal growth media, served as a standard with a REA=1.

Analysis of PSMA relative enzymatic activity revealed that there was an apparent increase for whole-cell protein samples for 2 days (REA = 1.26) and 5 passages (REA = 1.98), compared to the membrane-protein sample from normally-cultured LNCaP cells (Fig. 4). However, there was no detectable PSMA enzymatic activity after 10 passages in prolonged androgen-deprived conditions, similar to that of PSMA-negative PC-3 cells (Fig. 4), and consistent with the PSMA expression (Figs. 2 and 3).

## Discussion

Our data suggest that long-term androgen-depletion may induce downregulation of AR and PSMA which may lead to a diagnostically and therapeutically elusive androgen-independent disease-state. These new data provide additional knowledge of androgen-independent prostate cancer progression compared to previous studies, which primarily focused on changes in AR ligand binding specificity that may result from gene structure changes (e.g., mutation, amplification, or spliced variant expression) or AR ligand-independent activation arising from alternative signal pathways that activate AR activity at the castration level of androgen (2,24). With respect to PSMA, it is thought that increased expression is correlated with prostate cancer progression, especially in recurrent, metastatic cancers after androgen deprivation therapy (25,26).

While increased or consistent AR levels have been reported in established androgen-independent LNCaP sublines (27,28), loss of AR expression has been detected in other LNCaP sublines (4). Although all androgen-independent LNCaP sublines have been derived from a parent androgen-sensitive LNCaP cell line, they have been established in different labs under various conditions such as passage number of starting parent LNCaP cells, culture conditions, and duration of propagation under androgen-deprived growth conditions, which may result in different characteristics. In addition, the parent androgen-sensitive LNCaP cell line is not a homogeneous population, but is a multiple hypotetraploid mixture including cells with 84, 86, 87 or more chromosomes (product description of CRL-1740, http://www.atcc.org/). Single or a combination of multiple factors may contribute to these contradictory observations on AR expression in these established LNCaP sublines. A recent microarray analysis on patient tissues revealed that strong AR expression was detected in benign prostate (83%), localized prostate cancer (100%), and lymph node metastasis (80%), but less (40%) in metastatic hormone-resistant prostate cancer (3). It was also noticed that two highly aggressive androgen-independent prostate cancer cell lines: DU145 and PC-3 are double-negative of AR and PSMA (29) and the loss of their AR and PSMA expression is due to epigenetic silencing by CpG island hypermethylation of their promoter regions (29–31). The DU145 and PC-3 lines were derived from brain and bone metastases of prostate cancer, whereas the AR(+), PSMA(+) LNCaP cell line was derived from a lymph node metastasis. The attributes of these three cell lines are consistent with the above clinical analysis for AR expression. Although the molecular basis for downregulation of AR and PSMA expression still remains unclear, the present study reveals a dynamic progression in the loss of AR and PSMA expression during androgen deprivation. We also observed that LNCaP cells gained the capability of suspension growth and more rapid proliferation after 10 passages in culture with 10% charcoal-stripped fetal bovine serum and RPMI-1640 media (data not shown). Combined, the data suggest that the protein expression levels of AR and PSMA may correlate to the progression and metastatic sites of prostate cancer. Also, highly metastatic, aggressive, and androgen-independent prostate cancer cells are more likely to exhibit an AR(−) and PSMA(−) genotype. While some evidence is suggestive of novel roles for AR and PSMA as tumor-suppressor activity in androgen-independent prostate cancer cells (2,32), the loss of AR and PSMA expression may be a consequence of dedifferentiation or a stem cell-like transition of prostate cancer cells (33) induced by androgen deprivation.

It was observed that short-term androgen deprivation (up to 5 passages) lead to an apparent increase of PSMA enzymatic activity (Fig. 4) while cell labeling with a fluorescent PSMA inhibitor decreased for these time-points (Fig 2). This inconsistency may implicate a possible specific post-translational modification change (e.g., *N*-glycosylation pattern), which occurs during short-term androgen deprivation resulting in a modified PSMA with improved enzymatic activity and a relative loss of either affinity for inhibitors or cell-surface expression. In previous studies with cell lines and patient samples, different *N*-glycosylation patterns for PSMA have been detected (34,35). Furthermore, it has been confirmed that the different N-glycosylation patterns can strongly affect PSMA enzymatic activity (34,36). In addition, changes in substrate specificity and enzymatic activity were exhibited by brain GCP II (37,38), which shares sequence homology with PSMA (39), but is expressed in a different tissue.

In conclusion, our *in vitro* data suggest that the continuous, long-term androgen deprivation induces downregulation or loss of AR and PSMA expression in prostate cancer cells, which may have significance in the progression toward a more aggressive, metastatic prostate cancer disease-state during androgen deprivation therapy. The implication of these observations is that it may be critical to identify AR(+) or PSMA(+) tumors early to ensure the therapeutic efficacy of novel AR- or PSMA-targeted agents to treat recurrent, metastatic, and hormone-refractory prostate cancer patients. However, an unsolved question remains whether these androgen-independent LNCaP cells originate from the minor population of stem-like cancer cells or result from the accumulation of the changes in genetic structures, gene expression profiles, or alternative signal pathways in androgen-sensitive LNCaP cells.
